# Magnetic resonance imaging findings in juvenile spondyloarthropathy and effects of treatment observed on subsequent imaging

**DOI:** 10.1186/1546-0096-12-25

**Published:** 2014-07-11

**Authors:** Clara Lin, John D MacKenzie, Jesse L Courtier, Jeffrey T Gu, Diana Milojevic

**Affiliations:** 1 Children’s Hospital Colorado Pediatric Rheumatology, 13123 East 16th Street, Box B311, 80045 Aurora, CO, USA; 2 UCSF Pediatric Radiology, 505 Parnassus Ave, Moffitt Room: M396, Box 0628, 94143 San Francisco, CA, USA; 3 Floating Hospital for Children @ Tufts Medical Center, 800 Washington Street #190, 02111 Boston, MA, USA

**Keywords:** Spondyloarthopathy, Sacroiliitis, MRI, Enthesitis-related arthritis

## Abstract

**Background:**

Magnetic resonance imaging (MRI) is often used to diagnose and monitor treatment effects of juvenile spondyloarthropathy (SpA). Our objective was to describe MRI findings in juvenile SpA and determine predictors of active sacroiliitis and response to treatment.

**Methods:**

Children who had MRI of the sacroiliac (SI) joints and were referred to the pediatric rheumatology clinic from 2009 to 2012 were retrospectively studied. The clinical parameters, laboratory studies and findings on MRI were collected and a composite score ratio (CR) was calculated for both SI joints on each MRI study based on a semi-quantitative scale that included evaluation of bone marrow edema (BME), synovial enhancement (SE), and erosions (ER). The findings on MRI were correlated with clinical and laboratory values.

**Results:**

50 subjects who underwent 76 MRI for suspected or known SpA were included in the study. Sacroiliitis was seen in 48 MRIs in 32 subjects. Of the subjects with sacroiliitis, mean age ± standard deviation was 13.7 ± 2.6 years, 71% were male and 41% were HLA B27 positive. SE without BME was seen in 31% cases of sacroiliitis. In subjects with sacroiliitis, 79% also had hip arthritis and 41% had enthesitis of the pelvic region on MRI. In 38% of subjects with sacroiliitis, physical exam was not indicative of sacroiliitis or hip arthritis. Longitudinal data were available for 13 subjects. Sacroiliitis on MRI improved in 9 subjects with the greatest improvement in MRI composite score ratio after initiation of etanercept therapy. CR improvement was due to improvement of BME and SE components, while the ER score remained the same or worsened in all but 1 subject.

**Conclusion:**

History, physical exam or laboratory data may not predict sacroiliitis in children. Magnetic resonance imaging plays a valuable role in the initial evaluation and later treatment monitoring of children with spondyloarthropathy. Synovial enhancement is significantly reduced after treatment, and unlike adults, synovial enhancement may be detected without accompanying bone marrow edema, which suggests gadolinium contrast may be an important component in the assessment of children with spondyloarthropathy.

## Background

Spondyloarthopathy (SpA) is a type of arthritis with unique features of axial joint disease and abnormal new bone formation [1,2]. Sacroiliitis, arthritis of the sacroiliac (SI) joint, is a characteristic finding of spondyloarthropathy [1]. Many patients subsequently develop ascending arthritis of the spine and eventually ankylosis (fusion) [3]. Only a subset of children with chronic arthritis is at risk of developing SpA, including children with enthesitis-related, psoriatic, reactive, and inflammatory-bowel-disease-related arthritis [4,5]. Juvenile SpA is diagnosed when the disease starts prior to age 16 years [6]. Children typically first present with enthesitis and lower extremity peripheral arthritis prior to developing axial pathology [4,5]. Sacroiliitis can be asymptomatic and difficult to evaluate on physical exam.

Radiography only detects structural damage that occurs late in the disease. Magnetic resonance imaging (MRI) will detect earlier stages of inflammation and lesions of sacroiliitis that are occult on radiography [7]. Bone marrow edema can be observed on MRI in normal radiographs (prior to radiographic changes) and may herald sites of later erosion; [8] however, for pediatric patients no published guidelines exist on when an MRI is indicated to evaluate for sacroiliitis or monitor disease activity [1].

The aims of our study are to describe MRI findings of juvenile SpA, determine predictors of active sacroiliitis from the history, physical exam, and laboratory findings, and describe treatment effects of sacroiliitis as depicted on MRI.

## Methods

### Patients

This was a retrospective chart and imaging review of children age 5 to 21 years who were seen in the Pediatric Rheumatology clinics from 2009 to 2012. All children had an MRI of the SI joints ordered by either the pediatric rheumatologist or referring provider for evaluation of the SI joints for suspected SpA. Longitudinal data were collected from subsequent MRIs performed in subjects with a diagnosis of sacroiliitis. Sacroiliitis was defined by the presence on the first MRI examination of synovial enhancement, bone marrow edema, and/or erosions (Please see a detailed description of the MRI scoring system below).

### History and physical exam

Data were collected by chart review on electronic and paper medical records including demographics (age, gender, and race) and medication history. Race and ethnicity were collected by self-report. Race categories included: African American, Asian, American Indian, Pacific Islander, White, or Other; ethnicity included Hispanic or non-Hispanic. Since this was a retrospective study, the history and physical exam were not performed on the same date as the MRI scan. The history and physical exam were collected from the pediatric rheumatology clinic visit nearest the MRI scan date within 6 months of MRI scan date (prior of after scan date); however treatment changes could not be made within the time period between the clinic visit and MRI scan date.

Data collected from the subject’s history included complaints of back pain, hip pain, and morning stiffness. The history was collected from a review of systems checklist on the patient intake form and extrapolated from the recorded history of the treating rheumatologist. Elements of the physical exam collected included active hip arthritis defined as pain on range of motion (POM) and limited range of motion (LOM), LOM only or POM only of the hips, tenderness to palpation over SI joints, and tenderness over the SI joint on pelvic compression with moderate pressure, and anterior spinal flexion measured by modified pediatric Schober’s exam [9,10]. The modified pediatric Schober’s test was performed by measuring the expansion of two points drawn on the back during flexion of the lumbosacral spine [9,10]. Briefly, a horizontal line was first drawn between the dimples of Venus. Then two points were placed in midline along the spine 10 cm above and 5 cm below the horizontal line. The distance between these two points was measured while standing upright and subsequently while the patient maximally flexed his/her hips by attempting to touch the floor with knees kept straight. An expansion of less than 6 cm during flexion of the lower spine was considered abnormal.

### Laboratory data

Laboratory data collected included white blood cell count (WBC), platelet count (Plt), erythrocyte sedimentation rate (ESR), C reactive protein (CRP), and human leukocyte antigen B27 (HLA-B27). Laboratory data had to be within 12 weeks of MRI scan date, and no treatment changes could be made within the time frame between the laboratory collection date and MRI scan date. Abnormal inflammatory markers were defined as: WBC greater than 15 × 10 [9]/L, Platelet count greater than 450 × 10 [9]/L, ESR greater than 20 mm/hour, or CRP greater than 6 mg/L.

### MRI data

MRI examinations were performed at either 1.5 Tesla with a Signa Twinspeed or 3 Tesla Discovery 750HD scanner (General Electric Medical Systems, Milwaukee, WI) with patients placed in supine position. The following pulse sequences were obtained in an oblique coronal orientation dedicated to the SI joints: short tau inversion recovery (STIR), T1-weighted, and T1-weighted fat saturated pre- and post-gadolinium (intravenous Gd-DTPA 0.1 mmol/kg body weight) contrast enhanced images. The slice thickness was 4-5 mm; field of view = 20 cm; matrix = 512 × 512 for the T1-weighted sequence and 256 × 256 for the STIR and fat saturated T1-weighted sequences.

MR images were evaluated by a board-certified pediatric radiologist with an extra-year of fellowship training in musculoskeletal radiology and six years of post-fellowship experience. A subset of 22 MRI exams were evaluated independently by another board-certified pediatric radiologist with an additional year of fellowship training in abdominal and pelvic imaging and three years of post-fellowship experience in the interpretation of imaging examinations of pediatric bones and joints.

### MRI scoring

The MRI findings of sacroiliitis were defined as described by Rudwaleit et. al. and included the presence of synovial enhancement, bone marrow edema, and/or erosions [11]. MRI examinations were scored using a semi-quantitative scale from 0-3 (0 = no abnormality, 1 = mild, 2 = moderate, 3 = severe) for each SI joint in three categories: synovial enhancement (SE), bone marrow edema (BME), and erosions (ER). The amount of involvement of the joint determined the score: less-than one-third of the joint = 1, between one-third and two-thirds of the joint = 2, and greater than two-thirds of the joint = 3. The grading system is a modification of a previously described grading system from an adult study [12]. The scores take in the unique structure of the pediatric SI joint: smaller size, increased red marrow, and increased amount of cartilage in the epiphyseal equivalent portion of the joint. A composite score ratio (CR) was calculated for each MRI study as the sum of scores (left and right scores for SE, BME, and ER, total maximum sum of score = 18) divided by the number of components scored (total number of components = 6 for left and right SI, BME and ER). Six MRI studies were obtained without contrast, thus SE was not measured and the total maximum sum of score = 12, given the total number of components = 4. The presence of other sites of inflammation in the pelvis were also identified and included hip arthritis, SI joint fusion, facet arthritis, and pelvic enthesitis.

### Analysis

Predictors of MRI-diagnosed sacroiliitis were determined using Fisher’s exact for categorical variables (e.g. race, gender, HLA-B27) and two-sample t-tests for continuous variables (e.g ESR, modified pediatric Schober’s measurement). Inter-reader agreement for MRI scoring was evaluated using Kendall’s tau and Kappa statistics. Treatment effects on MRI scores in longitudinal data were evaluated using non-parametric analysis with Wilcoxon matched pairs signed rank test. All statistical tests were performed with STATA 12.0 software for Windows (StataCorp LP, College Station, TX, USA).

## Results

### Demographics

Fifty subjects were included in the study and their demographics are summarized in Table 1. Thirty-two of the 50 subjects had sacroiliitis and 13 had repeat examinations to study longitudinally.

**Table 1:** Demographics of total population and subjects with (+) sacroiliitis

	**Total subjects (n = 50 subjects, 76 exams)**	**Subjects with sacroiliitis (n = 32 subjects, 48 exams)**
	**n (%)**	**n (%)**
Age (years)	Mean ± SD = 13.3 ± 2.9	Mean ± SD = 13.7 ± 2.6
5-8	3 (6)	1 (3)
9-12	17 (34)	11 (34)
13-16	26 (52)	17 (53)
>17	4 (8)	3 (10)
Gender		
Male	35 (70)	23 (71)
Female	15 (30)	9 (29)
Race		
White, non-Hispanic	33 (66)	21 (66)
African American	8 (4)	2 (6)
Asian	3 (6)	2 (6)
Hispanic	9 (18)	6 (19)
Other	3 (6)	1 (3)
HLA-B27 (+)	23 (46)	13 (41)
≥1 elevated inflammatory marker^†^	37 (49)	24 (50)
MRI (+) hip arthritis	23 (45)	19 (79)
MRI (+) pelvic enthesitis	17 (22)	15 (31)

^†^at time of MRI exam.

### History and physical exam

70% of subjects complained of back pain, 48% hip pain, and 78% morning stiffness. Modified pediatric Schober’s exam was abnormal in 32% of subjects, tenderness over SI joint on palpation or pelvic compression in 30%, and hip arthritis by physical exam in 34%.

### MRI

Seventy-six MRIs were performed in 50 subjects, and 48 MRIs showed sacroiliitis in 32 subjects. ER was seen in 48 exams in 28 subjects, BME in 44 exams in 30 subjects, and SE in 42 exams in 29 subjects. The CR ranged from 0 to 2.67. The inter-reader agreement of CR and individual BME, SE, ER scoring on the subset of exams was poor with 27-54% agreement (kappa = 0.1-0.3). However, inter-reader agreement on presence or absence of active sacroiliitis showed substantial agreement of 81.8% with kappa = 0.6 (95% Confidence interval (CI = 0.3-1.0, z = 3.0, p = 0.0012). Due to the low kappa we then re-evaluated the images and used consensus to make a final determination for the scores for each patient.

**Figure 1:**
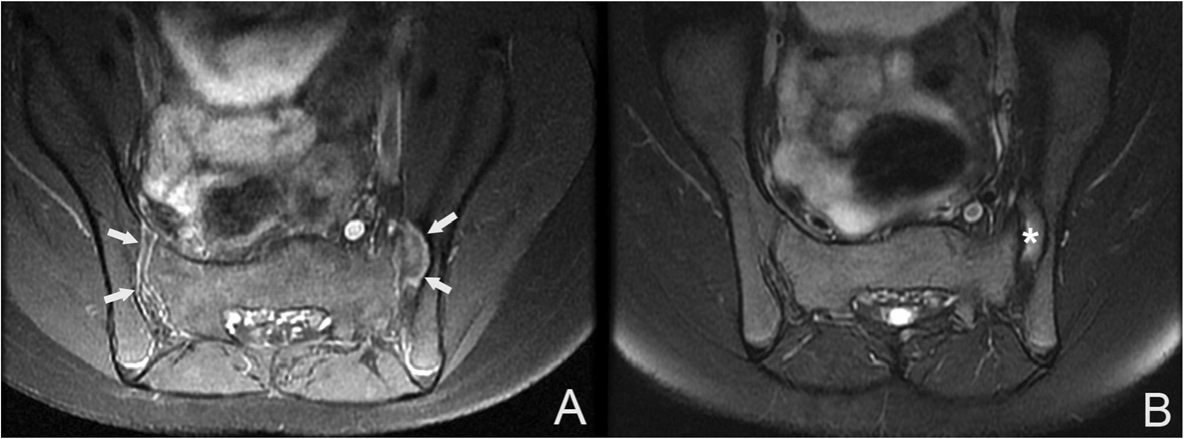
**Sacroiliitis without bone marrow edema.** Axial MRI images of the sacroiliac joints in an 8 year-old female with spondyloarthropathy shows bilateral synovial enhancement (arrows) and a large erosion in the left joint on the T1-weighted gadolinium contrast enhanced image **(A)**. Although the synovium in the erosion contains increased signal intensity (asterisk), no surrounding bone marrow edema is visible about either sacroiliac joint on the T2-weighted fat-suppressed image **(B)**.

Of the 32 subjects with MRI-diagnosed sacroiliitis, 71% were male (n = 23, male: female ratio = 2.6). Mean age was 13.7 ± 2.6 years (range: 8.2-20.8 years), and majority were White (65.6%) followed by Hispanics (18.8%) and African Americans (6.3%) (Table 1). HLA-B27 was present in 40.6% of subjects (n = 13) and unknown in 1 subject. Seven subjects had unilateral sacroiliitis, and 25 bilateral. Mean CR for subjects with sacroiliitis was 1.44 ± 0.59 and ranged from 0.33-2.67(maximum score = 3). Of the cases of MRI (+) sacroiliitis, BME was present in 91% of subjects and in 71% of SI joints with a score of 0 in 29%, 1 in 40%, 2 in 27%, and 3 in 4% of SI joints. Erosions were present in 91% of subjects and in 90% of SI joints with a score of 0 in 10%, 1 in 30%, 2 in 45%, and 3 in 15% of cases SE was present in all subjects and in 90% of SI joints cases where gadolinium was used with a score of 0 in 10%, 1 in 37%, 2 in 41%, and 3 in 12% of cases.There were 15 cases of sacroiliitis with SE score 1-2 without concomitant BME; of these, 12 had ER score 1-2 (Figure 1).

Hip arthritis could be evaluated in 28 MRI exams in 24 subjects with sacroiliitis and was present in 71.4% of exams in 79.2% of subjects (n = 19). Two subjects with sacroiliitis also had facet arthritis on MRI; one was a 14.7 year old HLA-B27 (+) Caucasian male, and the second a 14.4 year old HLA-B27 (-) African American female. Two subjects had partial fusion of the sacroiliac joint; one was a 16.8 year old HLA-B27 (+) Hispanic male, and the second a 19.3 year old HLA-B27 (+) Caucasian female.

We evaluated the history, physical exam, and laboratory tests at the clinic visit associated with MRI (+) sacroiliitis. Platelet count (p = 0.015, see Table 2) and MRI-diagnosed hip arthritis (p < 0.001) were the only significant predictors of sacroiliitis. In 67% of cases of sacroiliitis, subjects reported back pain. Using MRI as the gold standard for diagnosing sacroiliitis, sensitivity of an abnormal physical exam (tenderness to palpation over SI joint or pain on pelvic compression) to detect sacroiliitis was 22.9% and specificity was 67.9% when evaluating all 76 MRI scans. Sensitivity of an abnormal modified pediatric Schober’s exam was 36.4% (CI = 22.8-52.2%) and specificity 65.2% (CI = 42.8-82.8%) when evaluating all 76 MRI scans. To eliminate the issue of subjects contributing to more than 1 data point, we evaluated the sensitivity and specificity of an abnormal physical exam of the SI joint and modified pediatric Schober’s exam at only initial MRI of each subject. Sensitivity of an abnormal physical exam (tenderness to palpation over SI joint or pain on pelvic compression) to detect sacroiliitis was 30% (CI = 15.4-49.6%) and specificity was 72.2% (CI = 46.4-89.3%). Sensitivity of the abnormal modified pediatric Schober’s exam on was 44.4% (CI = 26-64.3%) and specificity was 73.3% (CI = 44.8-91.1%).

**Table 2:** Laboratory values in subjects with and without MRI (+) sacroiliitis

	**(+) Sacroiliitis (n = 48 MRIs, 32 subjects)**	**(-) Sacroiliitis (n = 28 MRIs, 18 subjects)**
Mean Laboratory results ± SD		
ESR	22.8 ± 22.8	21.9 ± 23.0
CRP	13.4 ± 30.6	4.7 ± 9.5
WBC	8.1 ± 2.7	8.8 ± 3.5
Platelets	357 ± 108	298 ± 98

Examining the history of the 32 subjects’ first MRI exams demonstrating (+) sacroiliitis, 75% reported back pain, 56% hip pain, and 78% morning stiffness at their associated clinic visit. Only 1 subject denied all 3 symptoms. Four subjects (13%) had a normal physical exam (normal modified pediatric Schober’s test, SI exam, and hip joint exam). No subjects were asymptomatic with a normal physical exam on their initial MRI demonstrating (+) sacroiliitis.

### Hip arthritis and pelvic enthesitis

The hip joints could be evaluated on 51 of 76 MRI exams in 41 subjects. Hip arthritis was found in 21 subjects on 23 MRI exams (45.1%) and associated with hip pain in 79.2% of exams. Concomitant sacroiliitis was seen in 87% of MRI in 90% of subjects. Using MRI as the gold standard, physical exam consistent with hip arthritis (abnormal LOM + POM) had sensitivity and specificity of 52.4% (CI = 30.3-73.6%) % and 64.3% (CI = 44.1-80.7%), respectively. When the presence of LOM or POM or both was used to diagnose hip arthritis, sensitivity was 87% (CI 65.3-96.6%) % and specificity 42.9% (CI 25-63.6%).

Enthesitis in the pelvic region was seen in 22.4% of all MRI exams and in 31.3% of MRI demonstrating sacroiliitis. Enthesitis was always associated with either sacroiliitis (n = 11 subjects) or MRI (+) hip arthritis (n = 2 = subjects).

### Longitudinal follow up

Fourteen of the 32 subjects with sacroiliitis had at least 1 subsequent MRI to monitor disease activity with a mean follow up period of 2.3 years (range: 0.3-7.0 years). The CR improvement was due to improvement of the SE component, while the ER score remained the same or worsened in all but 1 subject. The BME component did improve but was not statistically significant (Table 3). Interestingly, three of these subjects did not have sacroiliitis on their first MRI; of these, one was on no medication, and 2 were on non-steroidal anti-inflammatory drugs (NSAIDs) when sacroiliitis developed. CR improved in 9 subjects and was stable or worsened from initial MRI in 5 subjects.

**Table 3:** Changes in SI joint pathology on serial examinations

	**Initial MRI (mean ± SD)**	**Follow up MRI (mean ± SD)**	**p-value**
BME	2.4 ± 1.6	1.6 ± 1.5	0.16
ER	3.4 ± 1.4	3.6 ± 1.7	<0.0001
SE	3.5 ± 1.7	2.1 ± 1.4	0.04
CR	1.6 ± 0.7	1.3 ± 0.7	0.04

The mean combined score (right and left SI joint) for each component (BME, ER, SE) and the mean CR are shown for the first and follow up MRI (n = 14).

In 9 patients with improved CR, mean decrease in CR was 0.58 points (mean percent improvement = 38%). Improvement in composite score was due to improvements in BME and SE score components while the ER score component remained the same or worsened in all 9 subjects. HLA-B27 was present in 6 subjects and 8 subjects were male. Eight subjects were taking NSAIDs, 4 were taking etanercept (ETN) only, 3 were taking ETN + MTX, 1 was taking methotrexate (MTX) only, and 1 was taking sulfasalazine when CR decreased. ETN (including ETN + MTX) had the greatest mean% CR decrease of 38% compared to MTX only (25%), and sulfasalazine (20%). Interestingly, the 3 subjects on ETN + MTX had a lower mean %CR decrease (26%) compared to the 4 subjects on ETN only (47%).

Of the 5 subjects with worsened or stable composite score ratios, mean increase was 0.58 points (mean percent worsening = 18.9%). Worsening was due to worsening in all 3 components, greatest in the BME component (mean BME increase = 0.6 points). Erosion scores remained the same or worsened in all 5 subjects. HLA-B27 was present in 2 subjects, and 2 subjects were male. Two subjects were taking ETN + MTX, 2 NSAIDs only, and 1 MTX only when their composite score ratios worsened. Of the 2 subjects taking ETN + MTX, one subject was a 15 year-old HLA-B27 (-) female with bilateral sacroiliitis who had been on ETN for 3 months, and the second subject was a 14 year HLA-B27 (+) female with bilateral sacroiliitis who had been on ETN for 12 months.

Examining the history and physical exam during follow up MRI, 50% denied back pain, 44% hip pain, and 50% morning stiffness, and 31% denied all 3 symptoms despite persistent active sacroiliitis. On physical exam, modified pediatric Schober’s and hip and SI joint exams were normal in 8 subjects (50%) despite ongoing inflammation. Five subjects (31%) were asymptomatic and had a normal physical exam despite ongoing active sacroiliitis.

## Discussion

This work examines potential roles for MRI for children with SpA when placed in the context of their clinical history including physical examination, laboratory values, and treatment. Although this study has a relatively small cohort of 50 subjects with 32 subjects having sacroiliitis identified on MRI, we have found several important results and can draw some important conclusions. Synovial enhancement appears to be an important predictor of active inflammation and treatment effect.

The demographics of juvenile SpA differ from ankylosing spondylitis (AS). The male:female ratio was 2.6:1, which is significantly less than previously reported 7:1 ratio in juvenile AS [13]. This ratio is similar to recent studies in adult spondyloarthropathy (SpA) where the male: female ratio ranged from 2 to 5:1 [14-16]. HLA-B27 was present in 40.6% of the subjects with MRI (+) sacroiliitis, similar to a previous report by Pagnini et al. in 17 children with enthesitis-related arthritis (ERA) and sacroiliitis [17], but much less than reported in adult AS where HLA-B27 was present in >90% [18].

Predicting sacroiliitis in children from history, physical exam, or laboratory results remains a challenge. In our study, only elevated platelet count and MRI (+) hip arthritis predicted sacroiliitis. It is unclear if platelet count is a meaningful predictor, since other inflammatory markers (WBC, ESR, and CRP) were not predictive of sacroiliitis. Finding that hip arthritis as a predictor of sacroiliitis is consistent with previous studies in children with ERA or SpA which report hip arthritis as a predictor of sacroiliitis [17,19]. Unfortunately, abnormal hip physical exam findings were not sensitive or specific to predict sacroiliitis.

At the initial diagnosis of MRI (+) sacroiliitis, almost all patients were symptomatic with back pain, hip pain, or morning stiffness (97%) or had an abnormal physical exam (87%). However, on follow up MRI of previously diagnosed MRI (+) sacroiliitis, symptoms and physical exam were often normal despite ongoing inflammation. Of subjects with persistently active sacroiliitis on subsequent MRI, 50% had a normal physical exam, and 31% were asymptomatic with a normal physical exam. Therefore, children with previously diagnosed sacroiliitis and on therapy can still have active inflammation without findings on physical exam or history. This suggests that physical exam and history should not be the sole guides for altering the therapy for sacroiliitis in children.

The MRI characteristics of sacroiliitis in children demonstrated unique features specific to children compared to sacroiliitis in adults. In the majority of cases of MRI (+) sacroiliitis, all 3 components of the MRI score (BME, SE, ER) were positive. Interestingly, there were 15 (31.3%) cases of sacroiliitis where BME was not seen concomitantly with SE, and 80% of these cases had erosions. In adult spondyloarthropathy, SE is rarely seen without BME in cases of active sacroiliitis; therefore, non-contrast imaging for evaluating sacroiliitis has been advocated [20,21]. The extra cost and time for contrast-enhanced imaging are also factors to consider when imaging children [21]. However, non-contrast imaging for inflammatory arthropathy is controversial and at least one other study has evidence to support its use in JIA for evaluation of synovitis [22]. Our findings suggest that gadolinium may have added benefit in diagnosing sacroiliitis in children since synovial enhancement without BME was seen in our pediatric cohort.

In children, hip arthritis and pelvic enthesitis are often seen concomitantly with sacroiliitis on MRI. There was a high prevalence of MRI (+) hip arthritis (79%) in children with MRI (+) sacroiliitis, even without signs of hip arthritis on history or physical exam. This suggests that in children at risk of SpA, SI and hip joint MRI should be performed concomitantly in order to evaluate for the extent of the disease. Pelvic enthesitis diagnosed by MRI may be a specific finding in SpA. Pelvic enthesitis was seen in 31% of MRI (+) sacroiliitis and always associated with either sacroiliitis or hip arthritis on MRI. This is similar to findings in adult spondyloarthropathy where pelvic enthesitis is frequently associated with active sacroiliitis [11].

In subjects with follow up MRI exams, we were able to evaluate response to treatment. 69% of subjects had an improvement of their MRI composite score ratio, and the largest absolute and percent CR decrease (0.58 points, 47.2%) occurred with initiating etanercept therapy. Improvements in MRI composite score ratio was seen in inflammatory components (BME, SE) whereas erosions remained stable or worsened in all cases except one.

We observed no significant difference in the pre- and post-treatment groups for BME score. This may be due to several factors. The pre- and post-treatment trend for the BME score was variable; although there was improvement in the mean BME score, several patients actually showed an increase BME score after treatment. BME is a reactive phenomenon that may also be seen when secondary osteoarthritis or mechanical changes about a joint are present [23-25]. These patients are susceptible to secondary osteoarthritis and mechanical changes, so bone marrow edema does not always indicate active inflammation. This suggests the potential added benefit of gadolinium in monitoring treatment effects of sacroiliitis in children. The effects of etanercept therapy in this study are similar to findings in adult spondyloarthropathy where anti-TNF therapy improves inflammatory lesions but does not improve structural damage in the SI joint. [26] The reason for persistent or worsening erosions after therapy is unclear. Therapy may have no effect or a slower effect on structural damage. Improvement in structural damage may not be seen as soon as BME or SE improvements, and a longer follow-up period may elucidate whether etanercept has an effect on SI joint erosions. The one subject with improvement of erosion score had the longest follow up period (7.0 years).

### Limitations

This is a retrospective study with a small number of subjects especially subjects with longitudinal follow up. Many clinical variables were not recorded for all subjects, and useful information such as inflammatory character of the back pain, family history of HLA-B27 related disease, uveitis, improvement in pain/stiffness after therapy, etc. were not consistently available in the clinical records. The indication for requesting an MRI by the ordering physician was not clearly recorded in the medical record and may have varied among providers. The scoring system used in this study has yet to be validated. Several scoring systems have been utilized to evaluate sacroiliitis on MRI in the adult population. The scoring system implemented in this study was a modification from a study previously described in adults [12]. We needed to modify the grading system to reflect the unique structure of the pediatric SI joint. For example, the adult scoring system relies on obtaining six coronal slices through the joint; children often have smaller joints that are contained on less-than six slices.

We observed a high percentage of erosions and synovial enhancement in our study cohort than has been reported in other studies focused on adults [27,28]. In addition to the possible background erosions in a control subjects as described by Weber et al. [29] and whole body MRI technique that may lower sensitivity [28], the high percentage of erosions and synovial enhancement may reflect a referral bias to pediatric rheumatology in our institution. Also, the low kappa we observed for inter-reader agreement may be due to the large degree of heterogeneity of the appearance of sacroiliitis on MRI as reported by Weber et al. [29]. Also, the degree of heterogeneity of the appearance of sacroiliac joints is likely higher than adults due in part to the increased cartilage content, red marrow and the phenomena of increased foci of T2-weighted signal intensity observed in the pediatric skeleton [30].

## Conclusion

MRI of the sacroiliac joints is often useful in the workup or ongoing evaluation of pediatric patients who are suspected or have SpA. The imaging findings of SpA may be present in children when the physical examination is negative and changes in synovial enhancement may reflect treatment effects. Also, the MRI findings are different in children than adults. Unlike adults, synovial enhancement without bone marrow edema is often present and this supports the use of gadolinium for contrast enhanced imaging to detect and characterize SpA in pediatric patients.

## Abbreviations

MRI: Magnetic resonance imaging; SI: Sacroiliac; BME: Bone marrow edema; SE: Synovial enhancement; ER: Erosions; HLA: Human leukocyte antigen; UCSF: University of California-San Francisco; MRI: Magnetic resonance imaging; POM: Pain on range of motion; LOM: Limited range of motion; WBC: White blood cell count; ESR: Erythrocyte sedimentation rate; CI: Confidence interval; CRP: C-reactive protein; CR: Composite score ratio; SD: Standard deviation; NSAIDs: Non-steroidal anti-inflammatory drugs; ETN: Etanercept; MTX: Methotrexate; SpA: Spondyloarthropathy; AS: Ankylosing spondylitis; ERA: Enthesitis-related arthritis.

## Competing interests

Clara Lin, MD: none. John MacKenzie, MD: none. Jesse Coutier, MD: none. Jeff Gu: none. Diana Milojevic, MD: none. The authors declare that they have no competing interests.

## Authors’ contributions

CL conceived of the study, participated in its design, data acquisition, data analysis, and data interpretation, coordinated the study, and drafted the manuscript. JM participated in the design, interpreted all radiographic imaging, and critical review of manuscript for intellectual content. JC assisted in the interpretation of radiographic imaging. JG assisted with data acquisition, study coordination, and study design. DM conceived of the study, participated in its design and data interpretation, and critical review of manuscript for intellectual content. All authors read and approved the final manuscript.

## Authors’ information

CL is an Assistant Professor of Pediatric Rheumatology at Children’s Hospital Colorado. JM is a board certified pediatric radiologist with one with an extra-year of fellowship training in musculoskeletal radiology and six years of post-fellowship experience. His research focuses on developing imaging techniques for the detection and treatment monitoring of autoimmune diseases of the bones and joints. JM is an Assistant Professor in Residence and Chief of Pediatric Radiology at the University of California, San Francisco. JC is a board-certified pediatric radiologist with an additional year of fellowship training in abdominal and pelvic imaging and three years of post-fellowship experience in the interpretation of imaging examinations of pediatric bones and joints. He is an Assistant Clinical Professor in Pediatric Radiology at the University of California, San Francisco. His research interests include technique optimization in pediatric body MR applications. DM is Chief of Pediatric Rheumatology at the Floating Hospital for Children at Tufts Medical Center.
